# Clinical characterization of patients with schizophrenia and 16p13.11 duplication: A case series

**DOI:** 10.1002/npr2.12334

**Published:** 2023-04-28

**Authors:** Hiroki Kimura, Itaru Kushima, Masahiro Banno, Toshiya Inada, Akira Yoshimi, Branko Aleksic, Norio Ozaki

**Affiliations:** ^1^ Department of Psychiatry Nagoya University Graduate School of Medicine Nagoya Japan; ^2^ Medical Genomics Center Nagoya University Hospital Nagoya Japan; ^3^ Department of Psychiatry Seichiryo Hospital Nagoya Japan; ^4^ Division of Clinical Sciences and Neuropsychopharmacology, Faculty and Graduate School of Pharmacy Meijo University Nagoya Japan; ^5^ Institute for Glyco‐core Research (iGCORE) Nagoya University Nagoya Japan

**Keywords:** 16p13.11 duplication, copy number variation, NDE1, schizophrenia, VPS13B

## Abstract

**Background:**

Chromosome 16p13.11 duplication is a well‐known genetic risk factor for schizophrenia (SCZ) (odds ratio = 1.84). However, no case reports focusing on patients with SCZ and 16p13.11 duplication have been published. Therefore, here, we report the detailed clinical cases of four patients with SCZ and 16p13.11 duplication who were identified in our previous whole‐genome copy number variant (CNV) study.

**Case Presentation:**

In the four patients with SCZ and 16p13.11 duplication detected by array comparative genomic hybridization, one patient was found to have treatment‐resistant SCZ and an additional pathogenic rare CNV. Two of the four patients in this study had environmental risk factors that may have been involved in the development of SCZ.

**Conclusions:**

The results of this case series suggest that a genetic cohort study would be useful for evaluating which genetic and environmental risk factors could better explain the variable expressivity of 16p13.11 duplication. Furthermore, this work could be useful for elucidating the pathophysiology of SCZ.

## Abstract 2

Chromosome 16p13.11 duplication is a well‐known genetic risk factor for schizophrenia (SCZ) (odds ratio = 1.84). Therefore, here, we report the detailed clinical cases of four patients with SCZ and 16p13.11 duplication who were identified in our previous whole‐genome copy number variant (CNV) study.

## Graphical abstract

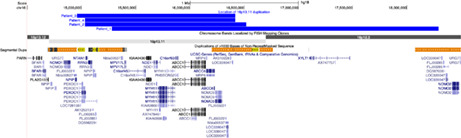

## 1 INTRODUCTION

Hundreds of genetic variants with a high effect size for psychiatric disorders have recently been identified, and genetic counseling based on these high‐effect variants has been performed. However, the detailed clinical course of carriers with these high‐risk variants remains unclear. Therefore, it is important to accumulate knowledge about carriers with high‐risk variants for psychiatric disorders to improve future genetic counseling.

Chromosome 16p13.11 duplication is one of the most well‐known risk factors for schizophrenia (SCZ)^1^ (odds ratio [OR] = 1.84).^2^ The pathophysiology of 16p13.11 duplication in SCZ is suggested to be derived from the genes related to the neurodevelopment in this region, such as nuclear distribution E homolog 1 (*NDE1*).^3^ However, to our knowledge, no case reports focusing on patients with SCZ and 16p13.11 duplication have been reported. Therefore, here, we report detailed clinical cases of four patients with SCZ and 16p13.11 duplication who were identified in our previous copy number variant (CNV) study.^4^

## 2 MATERIALS AND METHODS

### 2.1 Participants and phenotype evaluation

All patients were of Japanese ancestry and had been diagnosed with SCZ according to the Diagnostic and Statistical Manual of Mental Disorders, Fifth Edition.^5^ We retrospectively collected clinical data from the medical records of four patients with SCZ and 16p13.11 duplication. The data evaluated included developmental history, family history, academic career, professional career, medical history, psychiatric symptoms, age at onset of SCZ, history of hospitalization, medications, response to pharmacotherapy, comorbidity of physical disease, and other clinical manifestations. Treatment‐resistant schizophrenia (TRS)^6^ is defined as the persistence of symptoms despite receiving ≥2 antipsychotic medications of an adequate dose (≥600 mg/day chlorpromazine equivalent^7^) and duration (≥4 weeks at a therapeutic dosage) with documented adherence. Controls used for the expression analysis were selected from the general population and had no history of mental disorders based on responses to questionnaires or self‐reporting.

### 2.2 Genetic analysis

Genomic DNA was extracted from blood samples. CNVs in the 16p13.11 region were identified in four patients with SCZ using two types of array comparative genomic hybridization: NimbleGen 720 k Whole‐Genome Tiling Arrays (Roche NimbleGen) and Agilent SurePrint G3 Human CGH 400 K (Agilent Technologies).^4^ We generated CNV calls using Nexus Copy Number software, v9.0 (BioDiscovery). All genomic locations are given in hg18 coordinates.

### 2.3 Expression analysis of the NDE1 gene in LCLs

To investigate the effect of 16p13.11 duplication on *NDE1* mRNA transcription, we compared the relative expression of a patient (Patient 2) and 16p13.11 duplication with 55 healthy controls (40.5 ± 11.8) using lymphoblastoid cell lines (LCLs). LCLs were established by Epstein–Barr virus transformation of lymphocytes and cultured in RPMI‐1460 medium containing 20% fetal bovine serum, penicillin, and streptomycin. Total RNA was extracted from LCLs using the RNAqueous Kit (Invitrogen) and treated with DNase using the TURBO DNA‐free™ Kit (Invitrogen), then reverse‐transcribed to cDNA using the high‐capacity RNA‐to‐cDNA Kit (Invitrogen). Two housekeeping genes, hypoxanthine‐guanine phosphoribosyltransferase (*HPRT*) and glucuronidase‐beta (*GUSB*), were selected as internal control genes to normalize the polymerase chain reaction (PCR) tests. Real‐time quantitative PCR was performed with the probes of the predesigned TaqMan Gene Expression Assay (Hs00214339_m1for NDE1, Hs99999907_m1 for B2M, and Hs99999908_ml for GUSB; Applied Biosystems) using a real‐time PCR instrument (7900HT; Applied Biosystems). Measurement of the cycle threshold was implemented in duplicate. The data, including the amplifying efficiency and relative expression on quantification, were analyzed using the 2^–ΔΔ^ *C*_T_ method.

### 2.4 Case presentations

**FIGURE 1:**
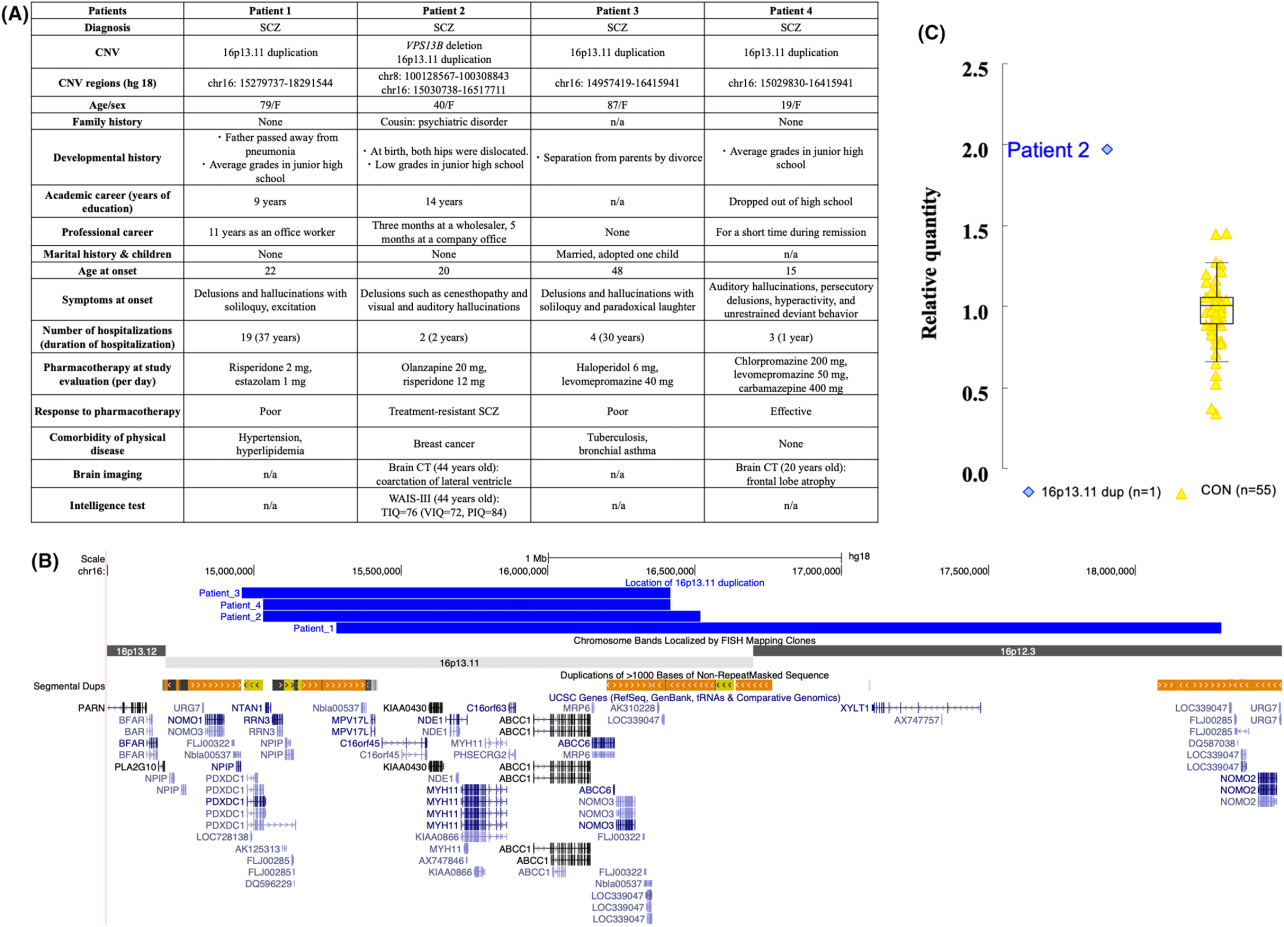
Summary of patients with SCZ and 16p13.11 duplication. (A) Clinical information of the four patients with SCZ and 16p13.11 duplication. The positions of the 16p13.11 duplications and *VPS13B* deletion were determined with reference to the following ensemble transcription ID based on NCBI Build (GRCh36/hg18). WAIS; Wechsler Adult Intelligence Scale, VIQ; verbal IQ, PIQ; performance IQ, TIQ; total IQ, CT; computed tomography. (B) Schematic representation of the 16p13.11 duplications identified in the four patients with SCZ. The 16p13.11 duplications identified in this study are shown in blue using the UCSC genome browser (https://genome.ucsc.edu). Segmental duplications, which facilitate nonhomologous recombination, are also shown. (C) The results of expression analysis of *NDE1*, which is located in the 16p13.11 region. We compared the relative expression of a patient with SCZ and 16p13.11 duplication (Patient 2) with 55 healthy controls using lymphoblastoid cell lines. Box plot: the box represents the middle 50% of observations. The middle bold line represents the median gene expression. Whiskers represent the minimum and maximum observations. Each dot represents the relative expression of each sample calculated by the 2^–ΔΔ^ *C*_T_ method. The orange dots represent the relative expression of controls (CON). The blue dot represents the relative expression of the carrier (Patient 2) with 16p13.11 duplication, and was higher than the first quartile of healthy controls. The relative quantity of 16p13.11 dup (*n* = 1) was 1.98, and the median (interquartile range) of CON (*n* = 55) was 1.00 (0.16).

Figure 1 summarizes the location of CNVs detected by array comparative genomic hybridization^4^ and the patients' clinical information.

Patient 1 was a 79‐year‐old female with hypertension and hyperlipidemia. At age 7 years, her father passed away from pneumonia. Her grades at junior high school were above average. After graduating from junior high school, she was employed as an office worker. At age 22 years, she developed delusions and hallucinations with soliloquy and excitation. Subsequently, she was diagnosed with SCZ and hospitalized repeatedly. At the time of this study, she had been hospitalized for 30 years from age 49 years. Due to her negative symptoms, such as abulia and social withdrawal, she did not engage with other patients in the hospital. At study recruitment, she was prescribed risperidone (2 mg/day) and estazolam (1 mg/day). However, her response to these psychotropic drugs was poor.

Patient 2, a 40‐year‐old female, had not only 16p13.11 duplication, but also vacuolar protein sorting 13 homolog B (*VPS13B*) deletion (Figure 1A). *VPS13B* is a risk gene for neurodevelopmental disorders.^8^ This patient had congenital bilateral hip dislocations, and her academic grades were low from elementary school through high school. After graduating from a 2 years college, she worked at a wholesaler for 3 months. At age 20 years, she developed auditory hallucinations and cenesthopathy and was diagnosed with SCZ. Because several types and amounts of antipsychotic medications failed to improve her symptoms, she was diagnosed with TRS. She was hospitalized for more than 2 years because of active hallucinations and delusions. At study recruitment, her response to olanzapine (20 mg/day) and risperidone (12 mg/day) (chlorpromazine equivalent: 2000 mg/day) was poor, and antipsychotic treatment with clozapine was not used. Gene expression analysis revealed an increased level of *NDE1* mRNA in this patient's LCLs (Figure 1C).

Patient 3 was an 87‐year‐old female. At age 4 years, her parents were divorced, after which, she was reared by her relatives. She later married and adopted one child. At age 48 years, she developed delusions and hallucinations with soliloquy and paradoxical laughter and was diagnosed with SCZ. After onset, her symptoms worsened, and she displayed disorganized speech and conduct. Several types of antipsychotics failed to relieve these symptoms. She was hospitalized in a psychiatric ward for more than 30 years with abulia and social withdrawal. Although she was prescribed haloperidol (6 mg/day) and levomepromazine (40 mg/day) at study recruitment, her response to antipsychotics was poor.

Patient 4 was a 19‐year‐old female. She was born by normal delivery and showed no abnormalities in growth or development. She had many friends and her junior high school grades were average. In the third grade of junior high school (age 15 years), she experienced auditory hallucinations, persecutory delusions, increased irritability, unrestrained deviant behavior, verbosity and hyperactivity, and insomnia. She was subsequently hospitalized and diagnosed with SCZ. Her psychiatric symptoms improved with treatment with antipsychotics. Although she was enrolled in high school, she refused to attend. After dropping out at age 18 years, she experienced hyperthymia and unrestrained behavior, followed by a manic state with verbosity, hyperactivity, extravagant behavior, and violence, leading to hospitalization. At study recruitment, she was prescribed levomepromazine (50 mg/day), chlorpromazine (200 mg/day), and carbamazepine (400 mg/day); this pharmacotherapy was effective for her psychiatric symptoms.

## 3 DISCUSSION

To our knowledge, this is the first case series focusing on patients with SCZ and 16p13.11 duplication.

Although 16p13.11 duplication is a well‐known risk factor for SCZ (OR = 1.84),^2^ it is not sufficient to cause SCZ. In other words, additional genetic or environmental factors may be involved in the development and severity of SCZ as a second hit.^9^

Patient 2 with TRS had 16p13.11 duplication and *VPS13B* deletion. Patients with SCZ with two pathogenic CNVs are more likely to show severe clinical manifestations.^10^ Considering that patient 2 had TRS, antipsychotic treatment with clozapine was chosen. NDE1 mRNA in the LCLs from patient 2 was high compared with the control samples. Regarding the effects of 16p13.11 duplication on the phenotypes of patient 2, we speculate that alterations in NDE1 dosage may affect brain development, as argued by Houlihan and Feng.^11^

In patients 1 and 3, environmental risk factors may have been involved in the development of SCZ. These patients experienced childhood adversities such as parental loss or separation. Such adversities are associated with a more than threefold increased risk of SCZ.^12^

Although 16p13.11 duplication has been reported to be associated with neurodevelopmental disorders (e.g., intellectual disability, autism spectrum disorder) and cardiovascular disease,^13^ no clinical information on these disorders was identified in this case series. This may be due in part to our limited access to health records. Moreover, the patients in this series may not be representative of individuals with 16p13.11 duplication because we only reported four patients with 16p13.11 duplication and SCZ. Therefore, a genetic cohort study would be useful for evaluating which genetic and environmental risk factors could better explain the variable expressivity of 16p13.11 duplication.^14^ Furthermore, to elucidate the molecular pathophysiology and drug development for SCZ, developing disease models such as induced pluripotent stem cells derived from carriers with 16p13.11 duplication would be useful.^15^ We believe that this work could be useful for elucidating the pathophysiology of SCZ.

## AUTHOR CONTRIBUTIONS

H.K., I.K., and N.O. designed the study. I.K. and A.Y. performed the genetic analysis. H.K., I.K., A.B., M.B., T.I., and N.O. recruited the participants and/or collected DNA samples or phenotype data. H.K. and I.K. wrote the first draft of the manuscript, and the other authors commented on and refined the manuscript. All authors carefully read the manuscript and approved the final version for submission.

## FUNDING INFORMATION

This research was supported by research grants from the Ministry of Education, Culture, Sports, Science and Technology of Japan (MEXT) and the Ministry of Health, Labour and Welfare of Japan; the Japan Agency for Medical Research and Development (AMED) under Grant Nos. JP21dm0207075, JP21ak0101113, JP21dk0307103, JP21ek0109488, JP19km0405216, JP22tm0424222, and JP21wm0425007; the Japan Society for the Promotion of Science (JSPS) KAKENHI Grant Nos. 15 K19720, 18H04040, 20 K20602, 21 K07543, 21H00194, 17H05090, 18 K19511, 21H02848, and 21H04815; the Uehara Memorial Foundation; and the SENSHIN Medical Research Foundation.

## CONFLICT OF INTEREST

H.K., I.K., M.B., A.B., and T.I. declare no conflict of interest. N.O. has received research support or speakers' honoraria from, or has served as a consultant to, Sumitomo Dainippon, Eisai, Otsuka, KAITEKI, Mitsubishi Tanabe, Shionogi, Eli Lilly, Mochida, DAIICHI SANKYO, Nihon Medi‐Physics, Takeda, Meiji Seika Pharma, EA Pharma, Pfizer, MSD, Lundbeck Japan, Tsumura, Novartis, Boehringer Ingelheim, Viatris, Kyowa, Janssen, Yoshitomi Yakuhin, Kyowa Kirin, Ono, Astellas, UCB, Taisho Toyama, Medical Review, and Woolsey outside the submitted work.

## ETHICAL APPROVAL

This study was approved by the ethics committee of Nagoya University Graduate School of Medicine and other participating institutes. Written informed consent was obtained from all participants. This study complied with all the provisions of the Declaration of Helsinki.

## REGISTRY AND THE REGISTRATION NO. OF THE STUDY/TRIAL

N/A

## Data Availability

In light of the ethical concerns surrounding the inclusion of detailed clinical information in this article, we make the raw data for CNVs identified in four patients available upon reasonable request to the corresponding author.
